# EBV-driven B-cell lymphoproliferative disorders: from biology, classification and differential diagnosis to clinical management

**DOI:** 10.1038/emm.2014.82

**Published:** 2015-01-23

**Authors:** Chi Young Ok, Ling Li, Ken H Young

**Affiliations:** 1 Department of Hematopathology, The University of Texas MD Anderson Cancer Center, Houston, TX, USA; 2 Department of Medical Oncology, University of Zhengzhou School of Medicine, Zhengzhou, China; 3 The University of Texas School of Medicine, Graduate School of Biomedical Sciences, Houston, TX, USA

## Abstract

Epstein–Barr virus (EBV) is a ubiquitous herpesvirus, affecting >90% of the adult population. EBV targets B-lymphocytes and achieves latent infection in a circular episomal form. Different latency patterns are recognized based on latent gene expression pattern. Latent membrane protein-1 (LMP-1) mimics CD40 and, when self-aggregated, provides a proliferation signal via activating the nuclear factor-kappa B, Janus kinase/signal transducer and activator of transcription, phosphoinositide 3-kinase/Akt (PI3K/Akt) and mitogen-activated protein kinase pathways to promote cellular proliferation. LMP-1 also induces BCL-2 to escape from apoptosis and gives a signal for cell cycle progression by enhancing cyclin-dependent kinase 2 and phosphorylation of retinoblastoma (Rb) protein and by inhibiting p16 and p27. LMP-2A blocks the surface immunoglobulin-mediated lytic cycle reactivation. It also activates the Ras/PI3K/Akt pathway and induces Bcl-xL expression to promote B-cell survival. Recent studies have shown that ebv-microRNAs can provide extra signals for cellular proliferation, cell cycle progression and anti-apoptosis. EBV is well known for association with various types of B-lymphocyte, T-lymphocyte, epithelial cell and mesenchymal cell neoplasms. B-cell lymphoproliferative disorders encompass a broad spectrum of diseases, from benign to malignant. Here we review our current understanding of EBV-induced lymphomagenesis and focus on biology, diagnosis and management of EBV-associated B-cell lymphoproliferative disorders.

## Introduction

Epstein–Barr virus (EBV) is a ubiquitous double-stranded DNA virus that belongs to the family Herpesviridae and subfamily Gammaherpesvirinae. Gammaherpesvirinae includes two important human gammaherpesviruses, EBV (also known as human herpesvirus 4) and Kaposi's sarcoma-associated herpes virus (also known as human herpesvirus 8 (HHV8)). EBV is a γ-1 herpes virus, characterized by a tropism for B-lymphocytes with latent infection in the host and the capacity for transforming B-lymphocytes. More than 90% of the population worldwide carry the virus. EBV was first discovered in a Burkitt lymphoma (BL)–derived cell line by Michael Epstein, Yvonne Barr and Bert Achong in 1964 by electron microscopy.^1^ Almost all EBV-seropositive hosts shed virus in the saliva, and infection occurs when an EBV-naive person is exposed to EBV-rich saliva.^2^ Oropharyngeal epithelium is an entry point for EBV via the immunoglobulin (Ig) A–EBV complex to IgA receptors on the epithelium, and active viral replication (lytic infection) occurs there.^3, 4^ The virus spreads out from the infected cells and directly infects nearby B-lymphocytes via viral enveloping of glycoprotein gp 350 to the B-lymphocyte surface molecule CD21.^5^ Penetration of the virus requires interaction between viral gp 42 (which makes a complex with gH and gL) and HLA-DR on B-lymphocytes.^6^ Indirectly, the infected epithelium can transmit the virus to B-lymphocytes. In infected B-cells, the linear genome circularizes and remains latent as episome in the nucleus (latent infection). Only a small proportion of the latently infected B-lymphocytes undergo viral replication spontaneously.

**Figure 1:**
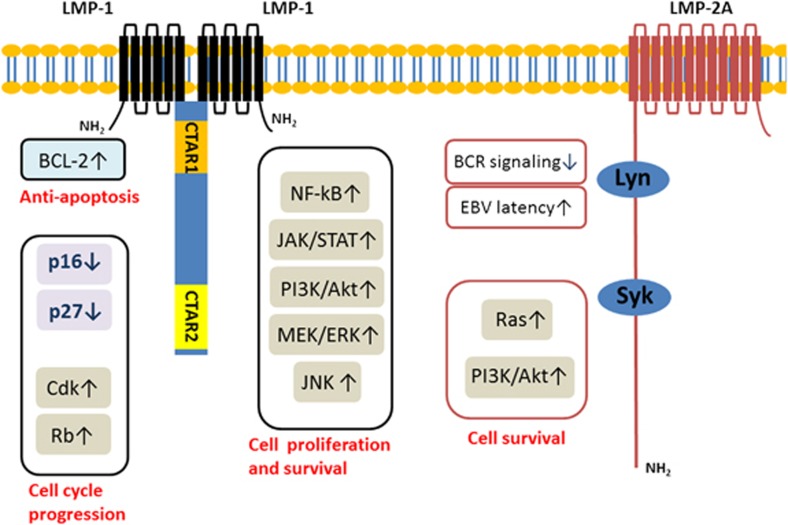
LMP-1 and LMP-2 and downstream signal transduction.

The EBV genome is a linear-shaped DNA of approximately 172 kb, encoding approximately 100 viral proteins. At both termini of the linear genome, there are variable numbers of 0.5-kb tandem repeats.^7^ In the lytic cycle, most of the EBV viral proteins are expressed but are kept in check by the host immune response. In contrast, only a handful of genes are expressed during latent infection: six EBV nuclear antigens (EBNA-1, 2, 3A, 3B, 3C and LP), three latent membrane proteins (LMP-1, 2A and 2B), and non-coding RNA (EBV-encoded small RNA (EBER)-1 and 2). EBNA-1 binds to viral DNA and maintains its episomal form.^8^ EBNA-2 transactivates LMP-1 via interaction with the cellular DNA-binding protein Jκ or PU.1.^9^ Because LMP-2B shares the EBNA-2 responsive promoter with LMP-1, EBNA-2 can also upregulate LMP-2B.^10^ Three different isoforms exist in EBNA-3, and they inhibit EBNA-2-mediated upregulation of LMP-1.^11^ EBNA-LP enhances the function of EBNA-2 to transactivate LMP-1. LMP-1 is oncogenic *in vivo* without expression of the other EBV gene and functionally mimics CD40, which is involved in B-cell activation and proliferation.^12, 13^ It is a six-transmembrane integral protein with a 200 amino-acid C-terminal cytoplasmic tail. This tail includes two important domains, C-terminal activation region 1 (CTAR1) and CTAR2. The transmembrane domain provides a platform for LMP-1 oligomerization, which results in constitutive activation of the molecule. Constitutively activated LMP-1 allows CTAR1 and CTAR2 to interact with downstream molecules, such as tumor necrosis factor receptor–associated factors (TRAFs) to activate the nuclear factor-kappa B (NF-κB) pathway.^14^ LMP-1 also activates the phosphoinositide 3-kinase (PI3K)/Akt pathway, c-Jun N-terminal kinase/AP-1, bcl-2, and A20, which inhibits p53-associated cell death.^15, 16, 17, 18, 19^ Cell cycle dysregulation can be caused by LMP-1 (Figure 1).^20^

LMP-2 has two isoforms, LMP-2A and LMP-2B. The genes for LMP-2A and LMP-2B span across the terminal repeats, so they are transcribed only when EBV is in circular form.^21^ They share 12 membrane-spanning domains and a 27 amino-acid C-terminal cytologic domain. Both LMP-2A and LMP-2B have EBNA-2 responsive promoters that have sites for RBP-Jk and PU.1, but their promoters are 3-kb apart.^10^ LMP-2A has an N-terminal cytoplasmic tail that binds to Lyn via SH2-binding motif and Syk via ITAM (immunoreceptor tyrosine-based activation motif).^22^ However, the N-terminal cytoplasmic domain in LMP-2A is lacking in LMP-2B.^23^ In B-lymphocytes, surface Ig (sIg)-mediated EBV lytic reactivation from latent infection can be induced via those protein tyrosine kinases.^24^ However, LMP-2A blocks the sIg-mediated lytic reactivation and therefore acts as a dominant-negative inhibitor of the sIg-associated protein tyrosine kinases. LMP-2A increases Bcl-xL expression and activates the Ras/PI3K/Akt pathway to mediate B-cell survival.^25, 26^ It also potentiates MYC-induced hyperproliferation by downregulating p27 in a proteasome-dependent manner.^27^ LMP-2B modulates LMP-2A *in vitro*, but its function *in vivo* is largely unknown.^28^ The function of EBERs is not clear, but EBERs are transcribed up to 10^7^ copies per cell in all forms of EBV latency.^29^ Currently, *in situ* hybridization for EBER is the most reliable method for morphological detection of latent EBV infection.^30^ On the basis of the latent gene expression, three different latency patterns are recognized. In type I latency, EBER and EBNA-1 are expressed. In type II latency, LMP-1, LMP-2A and LMP-2B are additionally expressed. In type III latency, all latent genes are expressed. Particular diseases with specific latency patterns are shown in Table 1.

Recent studies have expanded our knowledge of EBV microRNAs (miRNAs). EBV was the first virus in which miRNA was found.^31^ At the time of writing, 44 mature ebv-miRs have been recognized (Sanger miRBase library, release 20.0, http://www.mirbase.org/cgi-bin/query.pl?terms=EBV). Two independent ebv-miRNAs are known. The ebv-miR-BHRF1 family is located within the introns of BHRF1, and the ebv-miR-BART family is located in the introns of the BamHI-A region rightward transcript (BART) in two separate clusters.^32, 33^ Viral miRNAs can maintain viral latency, evade host immune response and inhibit lytic cycle reactivation.^34, 35, 36^ A recent study has shown that multiple cellular pathways, including p53 feedback loop, B-cell signaling, oxidative stress response and apoptosis pathways, could potentially be targeted by ebv-miRNAs.^37^ More specifically, ebv-miR-BHRF1 can promote cell cycle progression/proliferation and prevent apoptosis.^38^ ebv-miR-BART5 degrades a pro-apoptotic protein and p53-upregulated modulator of apoptosis.^39^ Cellular miRNAs can be dysregulated by EBV latent proteins. Mir-34a and mir-155 can be induced by LMP-1 through the NF-kB pathway.^40, 41^ EBNA-2 can enhance the NF-κB and Akt pathways by inducing miR-146a and downregulating miR-21, respectively.^42^

## Diagnosis, differential diagnosis and classification of EBV^+^ lymphoproliferative disorders

### Benign disorders

#### Infectious mononucleosis (IM) lymphadenitis

**Figure 2:**
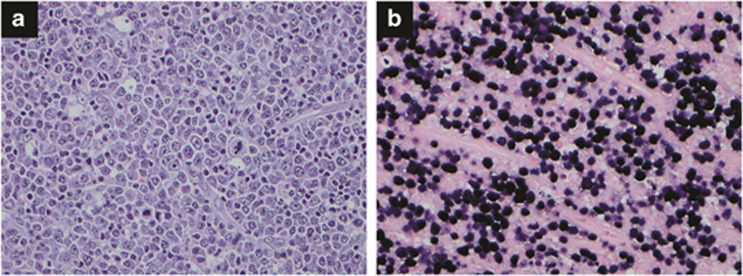
Infectious mononucleosis. (**a**) The paracortex of a lymph node is expanded by proliferation of immunoblasts. Hematoxylin and eosin, × 400. (**b**) *In situ* hybridization for EBER shows numerous positive cells, × 400.

IM lymphadenitis is an acute, self-limited lymphadenitis caused by EBV infection. In the United States, most young adults are exposed to EBV, and about half of them acquire IM. Patients generally have fever, pharyngitis, lymphadenopathy and splenomegaly. Complications are rare, but splenic rupture, upper airway obstruction, severe hematologic cytopenia or hepatitis can occur. Although complications happen, these symptoms can be resolved without sequelae. Results for the agglutination-based monospot test using horse erythrocytes are positive. EBV-specific antibodies such as IgM and IgG anti-viral capsid antigen antibodies or anti-EA antibodies appear, and circulating lymphocytes with abundant pale blue cytoplasm and large nucleus are increased. The lymph node architecture is partially effaced by paracortical hyperplasia, follicular hyperplasia or sinus histiocytosis. Paracortical expansion is the most common pattern, usually composed of a mixture of small lymphocytes, immunoblasts, plasma cells and histiocytes. Immunoblasts can form a sheet, mimicking diffuse large B-cell lymphoma (DLBCL) (Figure 2). By immunohistochemistry, CD20 highlights B-cell-rich follicles and B-immunoblasts in the paracortex. CD3 shows T-cells, mature lymphocytes and T-immunoblasts in the paracortex. *In situ* hybridization for EBER shows numerous EBV-infected cells. Ig genes and/or T-cell receptor genes show a polyclonal pattern.

#### EBV-positive mucocutaneous ulcer

EBV-positive mucocutaneous ulcer is a unique clinicopathological entity, first described by Dojcinov *et al.*,^43^ who reported it in 26 patients either with immunosuppression caused by azathioprine, methotrexate or cyclosporine A given for various autoimmune diseases or with age-related immunosenescence. The median patient age was 77 years (range 24–101 years), with a slight female predominance. Interestingly, all immunosuppressed patients who had available outcome data achieved complete remission when immunosuppressants were reduced. Patients with age-related immunosenescence showed spontaneous remission or relapsing and remitting clinical course when no therapy was given. All patients with age-related immunosenescence who underwent chemotherapy and/or radiotherapy achieved complete remission. Most importantly, none of the reported patients died of the disease irrespective of treatment.

This disorder occurs as isolated, well-demarcated ulcers in the oropharyngeal mucosa, skin and gastrointestinal tract. This lesion is characterized by a polymorphous infiltrate composed of lymphocytes, immunoblasts, plasma cells, histiocytes and eosinophils with atypical large B-cell blasts resembling Hodgkin Reed–Sternberg (HRS) cells. Plasmacytoid apoptotic cells were consistently observed in all cases, and angioinvasion was not uncommonly seen (23%). Immunohistochemistry shows a mixture of B- and T-cells in the polymorphic infiltrates. The HRS-like cells were of B-cell origin (CD20^+^, CD79a^+^, PAX5^+^, Oct-2^+^ and Bob.1^+^) with CD30 and CD45 expression in most cases. The intermixed T-cells were predominantly CD4-positive helper T-cells. *In situ* hybridization for EBER was positive in all cases, which accentuates demarcation of the lesion from the surrounding tissue. The EBER-positive cells co-expressed CD20 and PAX-5, showing the B-cell nature. Most cases showed at least phase II EBV latent infection with LMP-1 expression. Because of the presence of immunoblasts and HRS-like cells, classical Hodgkin lymphoma (CHL) or DLBCL can be misdiagnosed. Clonality tests might not help in distinguishing EBV-positive mucocutaneous ulcer from DLBCL or from T-cell lymphoma, because about 40% of cases showed clonal Ig gene rearrangement and T-cell receptor gene rearrangement, respectively.

### Gray disorders

#### Chronic active EBV (CAEBV) disease, B-cell type

CAEBV disease is an uncommon EBV-associated lymphoproliferative disorder affecting B-cells, T-cells or NK cells. It is currently defined as a severe progressive illness of >6 months that begins as a primary EBV infection or is associated with heavily elevated anti-EBV viral capsid antigen IgG ⩾1:5120 or anti-EBV early antigen IgG ⩾1:640 or markedly elevated EBV DNA in the peripheral blood with histological evidence (lymphocytic infiltration) of major organ involvement and increased EBER or proteins in the affected tissues in patients without an immunosuppressive condition.^44^ It was first described by Straus as ‘the chronic mononucleosis syndrome' to differentiate this disease from chronic fatigue syndrome.^45^ The major organ involvement denotes lymphadenitis, persistent hepatitis, splenomegaly, interstitial pneumonia, hypoplasia of the bone marrow or uveitis. The etiology of CAEBV is uncertain.^46^ EBV stimulates secretion of a variety of cytokines and chemokines in both EBV-infected cells and non-infected cells. The current recommendation is to specify the nature of the EBV-infected cells as being B-cells, T-cells or NK cells. In this review, we focus only on CAEBV, B-cell type. For T-cell or NK cell types, please consult the article by Kimura *et al.*^47^

Most cases of CAEBV have been reported in Asia, and almost all of the Asian cases have been of T-cell or NK cell origin.^47^ Unlike for Asian CAEBV, the most common type (57.9%) of CAEBV in United States is of B-cell origin.^44^ Of the 11 reported cases, 7 were men and 4 were women, with a median age at onset of 20 years (range 5–51 years). The ethnic distribution was eight white patients, one black, one Hispanic and one Asian. The most common signs and symptoms were lymphadenopathy (100%), followed by splenomegaly (72.7%), hypogammaglobulinemia (54.5%), fever and hemophagocytosis (45.5%), hepatitis (36.4%), hepatomegaly, central nervous system disease, peripheral neuropathy, interstitial pneumonia and pancytopenia (27.3%). Histological findings of CAEBV were not characteristic. Instead, prominent T-cell infiltrates and fewer numbers of EBER^+^ and CD20^+^ lymphocytes were seen in the affected tissues. Clonal rearrangements of the IgH genes were found in five (62.5%) of the eight tested cases. Anti-EBV viral capsid antigen IgG titer was elevated (⩾1:5120) in 50% of the 10 tested cases. More than half (56%) of the patients had decreased number of CD19^+^ B-cells compared with healthy individuals. Cytokine levels of interleukin-6, interleukin-10, tumor necrosis factor-α, and interferon-γ were significantly elevated in CAEBV patients compared with control patients. The 5-year overall survival rate for patients with B-cell CAEBV was 73, but 64% of the patients eventually died.

#### HHV8- and EBV-associated germinotropic lymphoproliferative disorder

HHV8- and EBV-associated germinotropic lymphoproliferative disorder is a very rare disease that was first described by Du *et al.*,^48^ reporting three patients. HHV8 latency-associated nuclear antigen (LANA) encoded by viral ORF 73 and EBER are defining features of this lesion, but the pathogenetic role of these viruses has not been fully investigated. Histologically, lymph node architecture is partially effaced with nodular proliferation of plasmablasts or bizarre anaplastic cells. The plasmablasts are not found in the mantle zone or the interfollicular area. The typical immunophenotype is CD10^−^, CD20^−^, CD30^+/−^, CD38^+/−^, CD79a^−^, CD138^−^, BCL2^−^, BCL6^−^, MUM1^+^, HHV8^+^ and EBER^+^. Expression of a monotypic Ig light chain indicates a B-cell origin of the disease. This lesion shows a polyclonal or oligoclonal pattern of Ig gene rearrangement and hence is considered a lymphoproliferative disorder and not a lymphoma. Although it shows favorable outcome after chemotherapy or radiotherapy, there is little information available to fully characterize its clinical behavior.

### Malignant disorders

#### Diffuse large B-cell lymphoma

**Figure 3:**
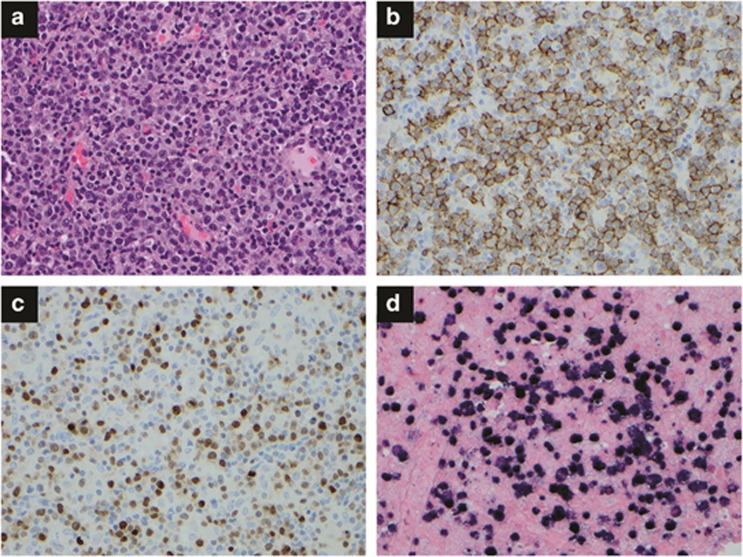
EBV-positive diffuse large B-cell lymphoma of the elderly. (**a**) The lymph node is completely effaced by a homogeneous population of large lymphoma cells. Hematoxylin and eosin, × 400. (**b**) CD20 stain shows numerous positive B-cells, × 400. (**c**) Ki-67 stain shows a high proliferation index, × 400. (**d**) *In situ* hybridization for EBER shows positive lymphoma cells, × 400.

EBV-positive DLBCL of the elderly. EBV-positive DLBCL of the elderly is defined as an EBV-positive monoclonal large B-cell lymphoproliferative disorder in immunocompetent patients aged >50 years.^49^ Cases of lymphomatoid granulomatosis (LyG), plasmablastic lymphoma (PBL), primary effusion lymphoma, DLBCL associated with chronic inflammation or IM are excluded. Table 2 summarizes the morphological, immunophenotypical and genetic findings in the major EBV^+^ lymphoid malignancies. The EBV-positive DLBCL of the elderly was originally described by Oyama *et al.*^50^ in 2003, emphasizing similarity to immunodeficiency-related lymphoproliferative disorder in immunocompetent elderly patients. An imbalanced immune system due to aging is postulated to promote lymphomagenesis. Of note, EBV positivity is generally determined to indicate positive expression of EBER in lymphoma cells, but a consensus for its cutoff has not been established (Figure 3).^51^ It is more common in Asian countries (8–11%) than in the Western hemisphere (2–4%), with variable EBER cutoffs.^52, 53, 54, 55, 56, 57^ Median patient age is 71 years (range 50–91 years), with a male to female ratio of 1.4 to 1. Epidemiological differences are seen in different geographic pockets. For example, EBV-positive DLBCL is more commonly reported in East Asia compared with Western countries. Mexican patients tend to be younger, to mostly have nodal involvement and to be less likely to express EBNA-2.^53^

World Health Organization (WHO) recognizes two main morphological subtypes, polymorphous and large-cell lymphoma (monomorphic). The polymorphous subtype demonstrates a range of B-cell maturation with a variable component of reactive elements. The monomorphic subtype shows a monotonous sheet of large transformed B-cells. Recently, Montes-Moreno *et al.*^58^ subdivided the polymorphous subtype into three further subgroups. However, morphological distinction does not indicate prognostic importance. The lymphoma cells express B-cell markers. CD30 is expressed in about 40% of cases. Expression levels of NF-κB component p50 and phosphorylated signal transducer and activator of transcription 3 (pSTAT3) are more commonly seen compared with EBV-negative DLBCL. LMP-1 and EBNA-2 are expressed in >2/3 and about 1/3 of cases, respectively, hence phase II or III latency patterns. Gene expression profiling showed that EBV-positive DLBCL is molecularly distinct from EBV-negative DLBCL. The gene set enrichment assay demonstrated an enhanced Toll-like receptor signaling pathway (which has many similarities to the NF-κB pathway) and the Janus kinase(JAK)-STAT pathway.^52^ Clonal rearrangement of the Ig gene is seen in most cases. Contrary to prior reports, mostly from Asian countries, of EBV-positive DLBCL with worse prognosis, a large cohort study from Western countries did not show adverse outcome in DLBCL with single expression of EBER. However, patients with co-expression of CD30 and EBER had a worse prognosis.^52^ Current treatment recommendation for DLBCL does not change for EBV positivity. EBV-positive DLBCL patients respond poorly and show variations to standard R-CHOP regimen with regard to ethnic background, CD30 expression, geographical distribution and oncogenic signaling activation. Adoptive immunotherapy against EBV latency antigens and implementation of oncogenic signaling target inhibitors targeting CD30, Btk or NF-kB components may improve outcome.

DLBCL associated with chronic inflammation. DLBCL with chronic inflammation is a B-cell neoplasm, mostly associated with EBV infection, arising in patients with a long-standing chronic inflammation, such as pyothorax, chronic osteomyelitis, metallic implant or chronic skin ulcer.^59^ The prototype of this lymphoma is pyothorax-associated lymphoma, first described in the English literature in 1987 by Iuchi *et al.*,^60^ who reported three cases during the period from 1971 to 1985. In an expanded series of 106 patients with pyothorax-associated lymphoma by the same study group, 93 (88%) cases were DLBCL, four cases (4%) were lymphoplasmacytic lymphoma and four cases (4%) were peripheral T-cell lymphoma. The immunophenotype could not be determined in five cases (4%).^61^ In this series, 80% of the patients had pyothorax resulting from artificial pneumothorax for the treatment of pulmonary tuberculosis, 17% had tuberculous pleuritis and 3% had pyothorax not associated with tuberculosis. Median age was 64 years (range 46–82 years), with a male-to-female ratio of 12.3:1. The median latency of developing DLBCL with chronic inflammation after pyothorax was 37 years (range 20–64 years). Common symptoms were chest or back pain, fever, tumor or swelling of chest wall, and respiratory symptoms such as productive cough with or without hemoptysis and dyspnea. The tumor is mostly confined to the pleura with invasion to the adjacent tissue, such as lung, diaphragm, ribs and mediastinum. Metastasis is uncommon. About half of the cases have large (>10 cm) tumor. Most cases are of lower (I/II) stage. Peripheral blood and bone marrow are usually spared.

Morphology is typical for DLBCL. The abutting pleural tissue shows extensive fibrous thickening with sparse inflammatory cells, including lymphocytes and plasma cells. Most lymphoma cells are positive for CD20 and CD79a but can be negative in cases with plasmacytic differentiation. MUM-1 and CD138 are positive in such cases. EBER and EBNA-2 are positive in most cases, illustrating a type III latency program.^61, 62^ Clonal rearrangement of the Ig gene is seen in most cases. Comparative genomic hybridization on pyothorax-associated lymphoma tumor samples demonstrated gain of chromosome 8q24, and *MYC* amplification was found by Southern blotting technique.^63^ Sequencing of the *TP53* gene (exons 5–8) using paraffin-embedded tissue found mutations in 2/3 of cases, with most being single-nucleotide substitution.^64^ By gene expression profiling, pyothorax-associated lymphoma was shown to be molecularly different from nodal DLBCL, with increased expression of activated B-cell-like signature.^65^ DLBCL with chronic inflammation is an aggressive lymphoma, with 1-, 3- and 5-year survival rates of 49, 27 and 22%, respectively.^61^

Plasmablastic lymphoma. PBL is an aggressive lymphoma with immunoblastic morphology but with plasmacytic immunophenotype.^66^ PBL was first described in 1997 by Delecluse *et al.*,^67^ who reported 16 cases mostly in HIV-positive patients. Since the initial description of exclusive occurrence in the oral cavity, PBL has been reported in the gastrointestinal tract, soft tissue, bone, gonads, mediastinum, anal canal and nasal/paranasal area.^68^ PBL was reported in patients without HIV infection or immunosuppression with common nodal involvement.^69, 70^ Median patient age is about 50 years (range 7–65 years), with a strong male predilection (7:1). Most patients are at advanced stage (stage III/IV) and with intermediate or high International Prognostic Index.

**Figure 4:**
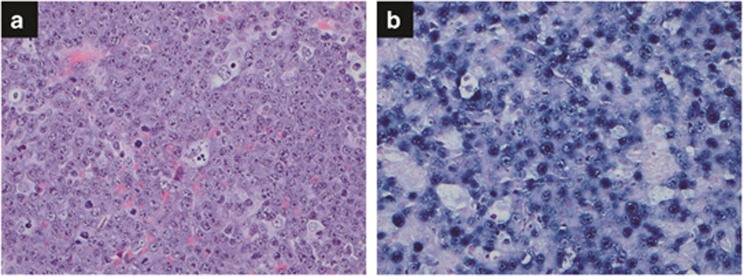
Plasmablastic lymphoma. (**a**) Sheets of immunoblasts and/or plasmablasts are present with occasional tingible-body macrophages, imparting a ‘starry-sky' pattern. Hematoxylin and eosin, × 400. (**b**) *In situ* hybridization for EBER shows numerous positive cells, × 400.

The lymphoma cells typically are of large size with round-to-oval centrally or eccentrically located nucleus, dispersed chromatin, prominent single nucleolus and amphophilic cytoplasm with perinuclear hof (Figure 4). Apoptotic cells with accompanying tingible-body macrophages can be seen, imparting a starry-sky pattern at low magnification. Mitotic figures are frequently seen, consistent with a high Ki-67 proliferation index (90–100%). The lymphoma cells mostly express plasmacytic markers such as CD38 (100%), MUM1 (100%) and CD138 (84%). CD79a is uncommonly expressed (14%); CD20 is virtually not expressed (3%). CD45 expression is seen in 1/3 of the cases. Frequent (80%) expression of epithelial membrane antigen is observed. HHV8 LANA is negative, which is very helpful for differentiating PBL from morphologically similar large B-cell lymphoma arising in HHV8-associated multicentric Castleman disease.^71^ Most PBLs are positive for EBER (78%), but with less frequent LMP-1 expression (38%). Hence, the latency pattern is either phase I or II. By FISH, *MYC* rearrangement was seen, with about 50% having *IgH* as the most common partner.^72^ Of note, *MYC* rearrangement was more commonly seen in EBER-positive PBL compared with EBER-negative PBL. Clonal rearrangement of Ig gene is seen in most cases. No effective treatment exists for PBL and patients often have a high stage at diagnosis and harbor a poor survival.

Primary effusion lymphoma. Primary effusion lymphoma (PEL) is a rare B-cell neoplasm presenting as a lymphomatous effusion in pleural, pericardial, or peritoneal cavities without detectable tumor mass, usually in immunosuppressant patients.^73^ The term PEL was first proposed by Nador *et al.* in 1996 and recognized by WHO in 2001 as a distinct clinicopathologic entity, although it had been recognized as a body cavity–based lymphoma.^74^ It is almost always associated with HHV8 and very commonly associated with EBV. Lymphoma with the same immunophenotype presenting as a solid tumor mass without malignant effusion is regarded as a variant of this entity and is called extracavitary PEL.^75^

Most patients are young or middle-aged homosexual men with HIV infection. PEL can arise in posttransplant patients without HIV infection or rarely in non-immunosuppressant patients.^76, 77^ HHV8 is believed pathogenetically to be more important than EBV in PEL, because HHV8 expresses oncogenic cellular homologues with a restricted pattern of EBV infection (mostly phase I).^78, 79^ Typical clinical presentation is effusion in a single body cavity without lymphadenopathy or organomegaly. Kaposi sarcoma precedes PEL in about half of the patients. Extracavitary PEL has been reported in lymph nodes and various extranodal locations, most commonly in the gastrointestinal tract.^80^

**Figure 5:**
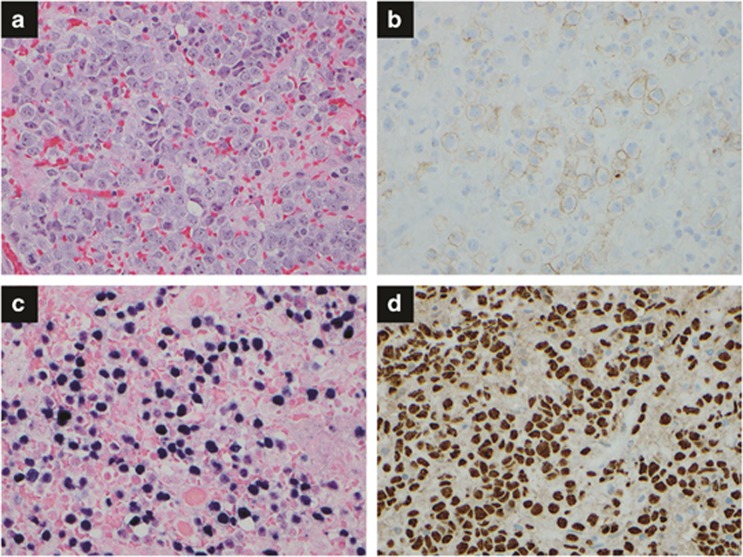
Extracavitary pleural effusion lymphoma. (**a**) Sheets of immunoblasts and/or plasmablasts are present with frequent mitotic figures. Hematoxylin and eosin, × 400. (**b**) CD138 stain is positive in most of the lymphoma cells, × 400. (**c**) *In situ* hybridization for EBER shows many positive cells, × 400. (**d**) HHV-8 stain is positive in the lymphoma cells, × 400.

In cytospin samples, the lymphoma cells show a morphological range from immunoblastic to anaplastic features. Variable numbers of large pleomorphic cells are seen, some of which resemble Reed–Sternberg cells. A prominent Golgi zone adjacent to the nucleus is often present in the lymphoma cells. In tissue sections, the lymphoma cells have round or ovoid to polygonal shape, moderate-to-large amounts of cytoplasm and round to variably indented nuclei with one or more prominent nucleoli (Figure 5). Multinucleated giant tumor cells and lymphoma cells with wreath-like nuclei, resembling hallmark cells in anaplastic large cell lymphoma, can be seen. Mitotic figures are numerous. The lymphoma cells usually express CD45 without expression of pan-B markers (CD19, CD20, CD22 and CD79a) or T/NK cell markers. Surface and cytoplasmic Igs are generally absent. CD30, epithelial membrane antigen, CD38, CD138 and HLA-DR are variably positive. LANA-1 of HHV8 is typically positive with a nuclear dot-like pattern. EBER is positive in about 70% of cases, but LMP-1 is negative. Extracavitary PEL shares a similar immunophenotype but with more common expression of B-cell markers.

Recurrent cytogenetic abnormalities have not been reported. Comparative genomic hybridization of eight PEL cases showed gain of chromosomes 12 and X in three and two cases, respectively, and amplification within the 1q region in two cases.^81^ *BCL-2*, *BCL-6* and *MYC* genes were not rearranged, and mutations in *MYC*, *HRAS*, *KRAS*, *NRAS* and *TP53* genes were not found.^74^ Clonal rearrangement of the Ig gene is seen in most cases and can be used for determining lineage. Gene expression profiling showed that PEL is distinct from non-Hodgkin lymphomas in immunocompetent patients and from AIDS-related lymphomas. The study also showed that PEL is in the differentiation stage of plasmablasts, because the gene expression profile showed features of immunoblasts, between EBV-transformed lymphoblastoid cell lines or AIDS immunoblastic lymphoma and plasma cells from multiple myeloma cell lines.^82^ Effective treatment lacks in PEL, and patients often demonstrate a dismal outcome.

Lymphomatoid granulomatosis. LyG was first reported by Liebow *et al.*^83^ in 1972 as nodular and granulomatous lesions. It is a rare EBV-driven angiocentric and angiodestructive lymphoproliferative disorder with a spectrum of clinical manifestation depending on the number of EBER-positive abnormal cells.^84^ It usually affects adults, typically in the fourth to sixth decade of life, with a male predominance (male-to-female ratio >2:1). Patients with congenital immunodeficiency, immunosuppressed patients or those with autoimmune disease are at increased risk. It is generally an extranodal disease. Lung involvement is almost always present, followed by kidney, skin and central nervous system. Lymph node and spleen are usually not involved at the initial presentation but can be involved when the disease has progressed. Lung involvement is usually bilateral, and middle/lower lobes are preferred sites. Clinically, it can present with fever, productive cough and dyspnea, mimicking infection. Other symptoms include malaise, weight loss, fatigue, variable neurological symptoms and gastrointestinal symptoms.

Histologically, LyG is characterized by a small number of EBER-positive intermediate-to-large atypical B-cells in a background of T-lymphocytes, plasma cells and histiocytes. Angioinvasion and angiodestruction are commonly seen. Although the name implies granulomatous reaction, well-formed granulomas are generally absent. Poorly formed granulomas can be seen in subcutaneous tissue.^85^ EBER-positive B cells have immunoblastic or pleomorphic morphology, resembling mononuclear Hodgkin cells. However, classic binuclear Reed–Sternberg cells are not present. The EBER-positive atypical B-cells are positive for CD20, with variable expression of CD30, but negative for CD15. CD4-positive T-cells are more predominant than CD8-positive T-cells in the background. Available data regarding latent EBV infection pattern in LyG showed latency II or III in most cases.^86^

LyG can be graded based on the number of EBER-positive atypical B-cells. Grade 1 LyG typically has only a few EBER-positive atypical B-cells (<5 per high-power field), and they might be absent in some cases. Grade 2 LyG typically has 5–20 EBER-positive atypical B-cells per high-power field, and grade 3 LyG usually has >50. Grade 3 LyG is usually regarded as a DLBCL equivalent because of its aggressive clinical behavior and therapeutic options similar to those for DLBCL. On the other hand, grade 1 and 2 LyG usually have an indolent course. However, one should be aware that about 15% of lower grade LyG can progress to malignant lymphoma.^87^ PCR analysis for Ig heavy chain gene rearrangement demonstrated a monoclonal pattern.^88^ Grade III LyG patients are treated with similar regimens used for DLBCL and DA-EPOCH-R has been reported to be effective with 66% complete remission. Interferon-α was reported to be effective in nearly 60% grade I and II LyG patients; however, a high frequency of recurrence is noted.

#### Burkitt lymphoma

BL is a germinal center–derived aggressive B-cell lymphoma with frequent *MYC* translocation. It was first described by Dr Dennis Burkitt in 1958 in Kampala, Uganda, as a common pediatric tumor involving the jaw.^89^ WHO currently recognizes three different clinical variants based on geographical information and HIV status.^90^ Endemic BL occurs in equatorial Africa and usually affects 4–7-year-old children, with a male predominance (2:1). Facial bones, especially jaw bones, are usually involved. Its geographical distribution overlaps with that of malaria infection.^91^ However, the role of malaria in the pathogenesis of BL is still unclear. Sporadic BL occurs worldwide, with a low incidence (1–2%) of all lymphomas. Young adults are usually affected, with a male:female ratio of 2–3:1. The ileocecal region is the most commonly affected site, followed by ovaries, kidneys and breasts. Immunodeficiency-associated BL specifically occurs in patients with HIV infection, congenital immunodeficiencies or posttransplantation status. All variants have a high tendency for central nervous system involvement. In endemic BL, EBV is detected in the majority of cases. However, EBV is seen in about 30% of sporadic and immunodeficiency-associated BL.

**Figure 6:**
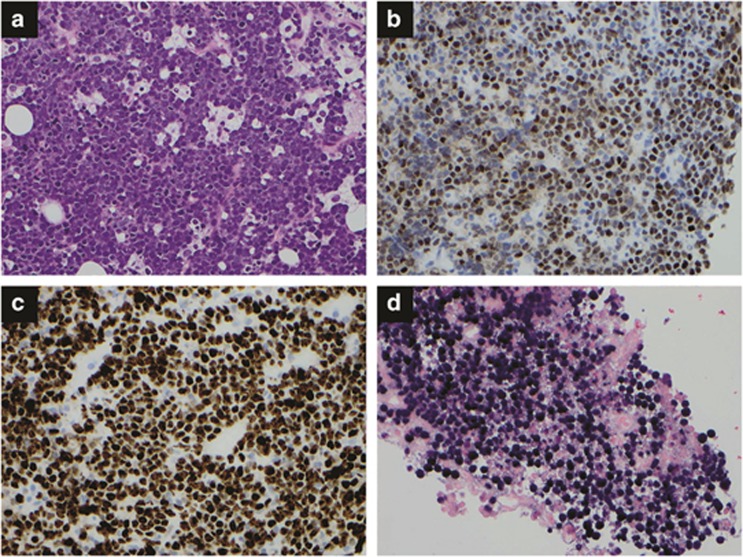
Burkitt lymphoma. (**a**) Sheets of medium-sized cells with numerous tingible-body macrophages showing the classic starry-sky pattern. Hematoxylin and eosin, × 400. (**b**) BCL-6 stain is positive in lymphoma cells, × 400. (**c**) Ki-67 proliferation rate is virtually 100%, × 400. (**d**) *In situ* hybridization for EBER shows numerous positive cells, × 400.

Endemic and sporadic BL typically shows a diffuse proliferation of intermediate-sized lymphoma cells, which have round-to-oval nucleus, dispersed chromatin, 2–3 discernible small nucleoli and squared-off borders for the cytoplasm. Numerous apoptotic cells with accompanying tingible-body macrophages are present, imparting a ‘starry-sky' pattern at low magnification (Figure 6). Rare cases might show an intense granulomatous reaction. In Giemsa-stained slides, lymphoma cells have basophilic cytoplasm with numerous cytoplasmic vacuoles. Immunodeficiency-associated BL commonly shows plasmacytic differentiation with eccentrically located nucleus, a prominent central nucleolus and more abundant cytoplasm. The lymphoma cells express B-cell markers, CD10 and BCL-6. BCL-2 expression is negative or weak and TdT is negative. The Ki-67 proliferation index is nearly 100%.

*MYC* rearrangement is characteristic but not a unique finding. t(8;14)(q24;q32) involving the Ig heavy chain region is commonly (80%) found, followed by t(2;8)(p12;q24) and t(8;22)(q24;q11) involving kappa and lambda light chain loci, respectively. Cytogenetic abnormalities other than *MYC* rearrangement are rare.^92^ Gene expression profiling revealed that BL closely resembles the centroblast-rich dark zone of the germinal center, whereas DLBCL with a germinal center B-cell-like phenotype is similar to the centrocyte-rich light zone of the germinal center.^93^ miRNA analysis found that expression of miRNAs targeted by Myc protein and miRNAs associated with NF-κB are significantly downregulated in BL compared with DLBCL.^94^ This study also showed that there was no or only minimal miRNA expression differences among the three BL variants, suggesting that the variants are the same biological entity with a different spectrum. Next-generation sequencing identified a novel loss-of-function mutation in *ID3*.^95, 96, 97^ The deleterious mutations disrupt the normal function of ID3 as a tumor suppressor and promote cell cycle progression and proliferation. Next-generation sequencing also revealed mutations in *MYC*, *TP53*, *DDX3X*, *TCF3*, *SMARCA4* and other genes. EBV^+^ BL patients show no difference in outcome from EBV^−^ BL, and EBV positivity in BL does not impact prognosis and treatment regimen selection. Patients commonly receive high-intensity, short-duration combination chemotherapy regimens, such as DA-EPOCH-R with nearly 100% response rate and low adverse event.

#### Classical Hodgkin lymphoma

Hodgkin lymphoma was first described by Sir Thomas Hodgkin in 1832.^98^ Hodgkin lymphomas include nodular lymphocyte–predominant Hodgkin lymphoma and CHL. CHL is further subdivided into nodular sclerosis, mixed cellularity, lymphocyte-rich and lymphocyte-depleted subtypes. CHL is characterized by the presence of scattered neoplastic HRS cells in a rich background of lymphocytes, eosinophils, plasma cells, neutrophils and histiocytes with various degrees of fibrosis. CHL has a peak of incidence in persons aged 15–35 years and a smaller second peak in an older group, aged 45–60 years. It often involves the axial lymph nodes, especially cervical nodes, and mediastinum with rare involvement of extranodal sites. CHL is usually a localized (stage I or II) disease, and the bone marrow is generally spared (<5%), except in patients with HIV infection (approximately 50%).

**Figure 7:**
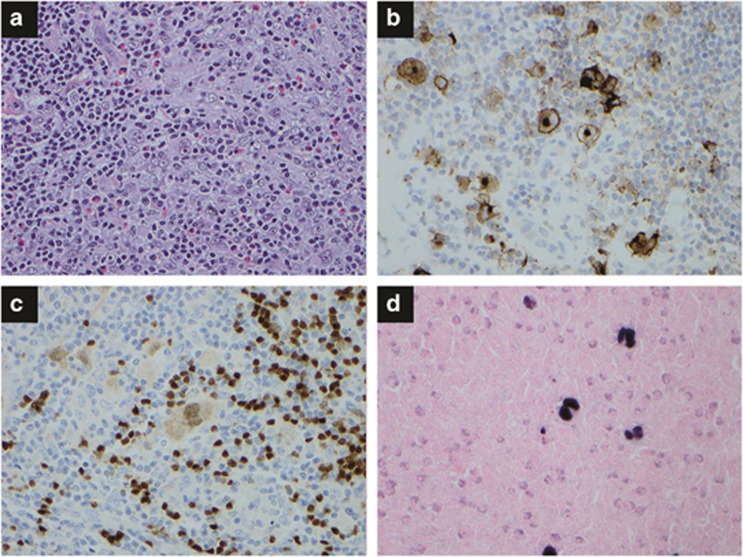
Classical Hodgkin lymphoma. (**a**) A classic binuclear Reed–Sternberg cell (center) and occasional mononuclear Hodgkin cells are intermixed with many mature-appearing lymphocytes, eosinophils, occasional plasma cells and histiocytes. Hematoxylin and eosin, × 400. (**b**) Hodgkin Reed–Sternberg cells express CD30 with a membranous and Golgi pattern, × 400. (**c**) A weak nuclear expression of PAX5 is seen in Hodgkin Reed–Sternberg cells. In contrast, B-lymphocytes in the background show strong expression of PAX5, × 400. (**d**) Occasional Hodgkin Reed–Sternberg cells are positive for EBER, × 400.

The classic Reed–Sternberg cells are large cells with two or more round nuclei, dispersed chromatin, prominent nucleolus and abundant basophilic cytoplasm with a perinuclear halo (Figure 7). The mononuclear variant is called the Hodgkin cell. HRS cells are characteristically positive for CD30, CD15, PAX-5 (weak/nuclear expression), MUM-1 and fascin but negative for CD3, CD20, CD45, CD68 (PG-M1), CD79a and CD138. EBV is seen in CHL, most commonly in mixed cellularity and lymphocyte depleted, followed by lymphocyte rich and nodular sclerosis. EBV-infected HRS cells usually express LMP-1 without EBNA-2 expression (phase II latency).^99^ HRS single-cell PCR showed a monoclonal Ig gene rearrangement with preservation of Ig-coding capacity, suggesting that the lack of B-cell markers is caused by defects in Ig gene regulatory elements.^100^ Array-based comparative genomic hybridization applied to microdissected HRS cells revealed frequent recurrent gains in chromosomes 2p12-16, 5q15-23, 6p22, 17q12 and 19p13 and losses in Xp21, 6q23-24 and 13q22.^101^ Gene expression profiling of microdissected HRS cells showed downregulation of B-cell lineage genes, deregulation of transcription factor networks and upregulation of NF-κB and JAK-STAT pathway–related genes and various chemokines and cytokines.^102^ A miRNA signature was found with 234 differentially expressed miRNAs in CHL.^103^ Gene expression profiling data and miRNA data could provide additional information on risk stratification of CHL. Primary EBV^+^ HL patients show a poor survival from EBV^−^ HL; however, EBV positivity does not impact treatment regimen selection. In recurrent EBV^+^ HL with worse prognosis, adaptive immunotherapy, CD30 antibody and NF-kB pathway inhibitors may show therapeutic promise.

#### Posttransplant lymphoproliferative disorders

Posttransplant lymphoproliferative disorders (PTLD) are a heterogeneous group of diseases in immunocompromised patients who are recipients of a solid organ or hematopoietic stem cell transplant.^104^ PTLD shows a spectrum from indolent polyclonal proliferation to overtly malignant proliferation of lymphocytic or plasma cells. About 5% of PTLD are T-cell type. PTLD was first described by Doak *et al.*^105^ in 1968 in two renal transplant recipients. The first year after transplantation has the highest risk.^106^ Up to 2/3 of PTLD cases are associated with EBV. The risk of developing PTLD is higher in EBV-negative recipients than in EBV-positive ones, with the highest risk in EBV^+^ donor/EBV^−^ recipients.^107^ An increase in EBV viral load and a decrease of EBV-specific cytotoxic T-cells are associated with the development of PTLD.^108^ Recipients of solid organ transplants, especially heart, lung, intestine or multiple organs, have higher risks of PTLD compared with patients with hematopoietic stem cell transplant. PTLD involves extranodal sites, including the tonsils, gastrointestinal tract, lung and liver as well as lymph nodes.

**Figure 8:**
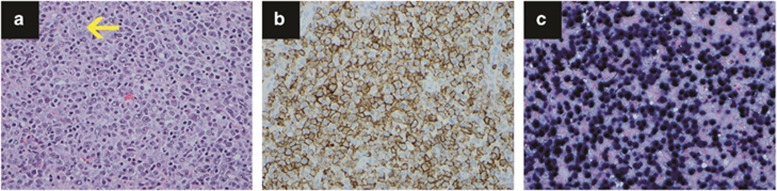
Posttransplant lymphoproliferative disorder, polymorphic type. (**a**) A mixture of immunoblasts, lymphocytes, plasma cells and Hodgkin Reed–Sternberg-like cells (arrow) is present. Hematoxylin and eosin, × 400. (**b**) CD20 stain shows a B-cell proliferation, × 400. (**c**) *In situ* hybridization for EBER shows numerous positive cells, × 400.

Morphologically, PTLD can be categorized by early lesions, polymorphic PTLD, monomorphic PTLD and CHL-type PTLD. Early lesions are further subdivided into plasmacytic hyperplasia and IM-like lesions. Plasmacytic hyperplasia is characterized by an increased number of plasma cells without effacement of tissue architecture and with residual germinal centers. IM-like lesions show the typical morphology of IM as described above. Both show minimal cytological atypia. Immunophenotype and molecular studies do not usually detect monotypic/monoclonal B-cell or plasma cell populations. Polymorphic PTLD is a destructive extranodal mass or a lesion with effacement of lymph node architecture. A polymorphic population of lymphocytes, plasma cells, immunoblasts and histiocytes is typically seen, with a variable presence of HRS-like cells (Figure 8). Of note, the full range of B-cell maturation is present, which is a morphological clue in differentiating polymorphic PTLD from monomorphic PTLD. Monotypia may or may not be seen, but the Ig gene is clonally rearranged. Monomorphic PTLD is an outright lymphoma, identical to that in immunocompetent patients. Of note, follicular lymphoma and mucosa-associated lymphoid tissue lymphoma are not designated as PTLD.^104^ CHL-type PTLD should have classic morphology and immunophenotype and should be differentiated from polymorphic PTLD with HRS-like cells. That is, the immunophenotype for CHL-type PTLD is CD15^+^, CD20^−^, CD30^+^, CD45^−^ and PAX5 weak^+^, whereas the immunophenotype for polymorphic PTLD with HRS-like cells is usually CD15^−^, CD20^+^ and CD45^+^. EBER is almost always positive. These categories represent a spectrum of disease and can be difficult to separate in clinical practice.

Conventional karyotypic studies are correlated with morphological categories. Clonal karyotypes were seen 15–30% of polymorphic PTLD and 70–75% of monomorphic PTLD.^109, 110^ Array comparative genomic hybridization detected gains at 5p13.1-p15.33 and 11p11.2-p15.5 and losses of 17p13.2-p11.2, 12p13.31-p13.3 and 12q24.33, with low frequency in monomorphic PTLD, DLBCL type.^111^ Gene expression profiling of PTLD showed a clear segregation between EBV^+^ PTLD and EBV^−^ PTLD, which had minimal differences from DLBLC in immunocompetent patients. In the former, upregulated genes were related to an innate immune response induced by interferon, NK and cytotoxic T-cell markers and tolerance.^112^ Data regarding epigenetics is rare, but hypermethylation of O^6^-methylguanine-DNA methyltransferase and death-associated protein-kinase was seen in 57% and 72% of PTLD, respectively.^113^ Proteomic analysis of monomorphic PTLD showed upregulated proteins in the PI3K/Akt, mitogen-activated protein kinase, protein kinase C and NF-κB pathways.^114^ PTLD typically shows type III latency features with expression of nine EBV latency proteins (Table 2), and no optimal management approaches exist. Reduction of immunosuppression may induce durable remission in localized or indolent disease but harbor risk for the graft rejection. Variable successes are observed in aggressive type treated with combination immunochemotherapy. Immunotherapy regimens with EBV-specific cytotoxic T-lymphocytes have shown promising efficacy for prevention and treatment.

Other immunomodulator-associated lymphoproliferative disorders have been rarely reported. Similar to PTLD, iatrogenic immunodeficiency-associated lymphoproliferative disorders have been reported in patients who had been treated with purine analogue (fludarabine, pentostatin or clofarabine) with or without alemtuzumab, methotrexate, anti-tumor necrosis factor-α agents (infliximab, adalimumab or etanercept) or other immunomodulators (daclizumab, anakinra or efalizumab) for various lymphoproliferative disorders, leukemia or autoimmune diseases.^115, 116, 117, 118, 119, 120^ Association with EBV was also seen in variable numbers of such cases. A broad range of clinical manifestation has been reported, ranging from regression without treatment to an aggressive course leading to patient death. Reduction or withdrawal of immunosuppression may induce durable remission in some patients.

## Summary

EBV is a well-known oncogenic virus and affects about 90% of the population worldwide. Once EBV infects B-lymphocytes, it achieves latent infection as an episomal form with limited expression of a few latent genes. EBV latent proteins are well known for oncogenesis, and EBV is associated with various lymphoproliferative disorders that range broadly from benign diseases to aggressive malignant neoplasms. Oncogenic role and mechanism that EBV has vary in different clinical setting, lymphoma subtype, ethnic predisposition and genomic aberration. Optimal treatment is generally lacking in most of EBV^+^ lymphomas; novel therapeutic approaches and associated randomized clinical trials for prevention and treatment are heavily demanded. Three latency types are associated with unique entities of lymphoma pathogenesis; type I relates to selective EBNA-1 expression in BL, PEL and PBL. Immunotherapy with EBNA-1-specific CD4^+^ cytotoxic T-lymphocytes is likely effective. Type II relates to the expression of EBNA-1, LMP-1 and LMP-2 in CHL and a subset of DLBCL. Cellular therapy targeting on LMP-2 may induce clinical response. Type III associates with the expression of nine EBV proteins in PTLD, IM, LyG and DLBCL. Future studies are valuable to explore novel therapeutic agents, including miRNA-targeted therapy, EBV vaccine, specific EBV signaling pathway inhibitors and combination of EBV lytic phase induction and anti-EBV drugs.

## Figures and Tables

**Table 1:** Epstein–Barr virus latency in selected EBV-driven lymphoproliferative disorders

*Latency*	*Gene products*	*Disease*
I	EBER, EBNA-1	BL, PBL, PEL
II	EBER, EBNA-1, LMP-1, LMP-2A, LMP-2B	CHL, EBV^+^ DLBCL of the elderly, PBL (subset), LyG (subset), PTLD (subset)
III	EBER, EBNA-1, LMP-1, LMP-2A, LMP-2B, EBNA-2, EBNA-3A, EBNA-3B, EBNA-3C	IM, DLBCL with CI, LyG, PTLD, EBV^+^ DLBCL of the elderly (subset)

Abbreviations: BL, Burkitt lymphoma; CHL, classical Hodgkin lymphoma; DLBCL, diffuse large B-cell lymphoma; DLBCL with CI, diffuse large B-cell lymphoma associated with chronic inflammation; EBER, Epstein–Barr virus encoded small RNA; EBNA, Epstein–Barr virus nuclear antigen; IM, infectious mononucleosis; LMP, latent membrane protein; LyG, lymphomatoid granulomatosis; PBL, plasmablastic lymphoma; PEL, primary effusion lymphoma; PTLD, posttransplant lymphoproliferative disorder.

**Table 2:** Morphologic, immunophenotypic and genetic findings helpful for differential diagnoses

*Disease*	*Morphology*	*Immunophenotype*	*Genetic*
EBV+DLBCL of the elderly	Polymorphic and monomorphic subtypes	CD20^+^, CD30^+/−^, EBER^+^, LMP-1^+^	*BCL2*, *BCL6*, *C-MYC* and *TP53* usually not rearranged
DLBCL with CI	Typical DLBCL morphology	CD20^+^, CD138^−^, but CD20^−^,CD138^+^ in cases with plasmacytic differentiation	*TP53* mutation
PBL	Sheets of immunoblasts or plasmablasts	CD20^−^, CD138^+^, MUM1^+^, EMA^+^, EBER^+^, HHV8^−^	*MYC* rearrangement
PEL	Immunoblastic or anaplastic cells in cytopsin, plasmablastic or immunoblastic in tissue section	CD45^+^, CD20^−^, CD79a^−^, sig^−^, CD30^+/−^, CD138^+/−^, CD38^+/−^, EMA^+/−^, HHV-8^+^, EBER^+^, LMP-1^−^	*BCL2*, *BCL6* and *C-MYC* usually not rearranged
LyG	Angioinvasion, angiodestruction, polymorphic background with several large atypical lymphoid cells. HRS-like cells present	CD20^+^, CD30^+/−^, CD15^−^, EBER^+^, LMP-1^−/+^	Monoclonal immunoglobulin heavy chain gene rearrangement in grade 2 or 3
BL	Starry sky pattern, sheets of intermediate-sized lymphoid cells with round-to-oval nucleus	CD10^+^, CD20^+^, BCL-2^−^, BCL-6^+^, Ki-67 100%	t(8;14), t(2;8), t(8;22)

Abbreviations: BCL, B-cell lymphoma; BL, Burkitt lymphoma; DLBCL, diffuse large B-cell lymphoma; DLBCL with CI, diffuse large B-cell lymphoma associated with chronic inflammation; EBER, Epstein–Barr virus encoded small RNA; EMA, epithelial membrane antigen; HHV8, human herpesvirus 8; LMP, latent membrane protein; LyG, lymphomatoid granulomatosis; MUM1, multiple myeloma oncogene 1; PBL, plasmablastic lymphoma; PEL, primary effusion lymphoma.
